# Optimization of membrane excitability for predictive homeostasis of spike generation

**DOI:** 10.1186/1471-2202-15-S1-P161

**Published:** 2014-07-21

**Authors:** Jaekyung K Kim, Christopher D Fiorillo

**Affiliations:** 1 Bio and Brain Engineering, KAIST (Korea Advanced Institute of Science and Technology), Daejeon 305-701 Korea

## 

**Figure 1:**
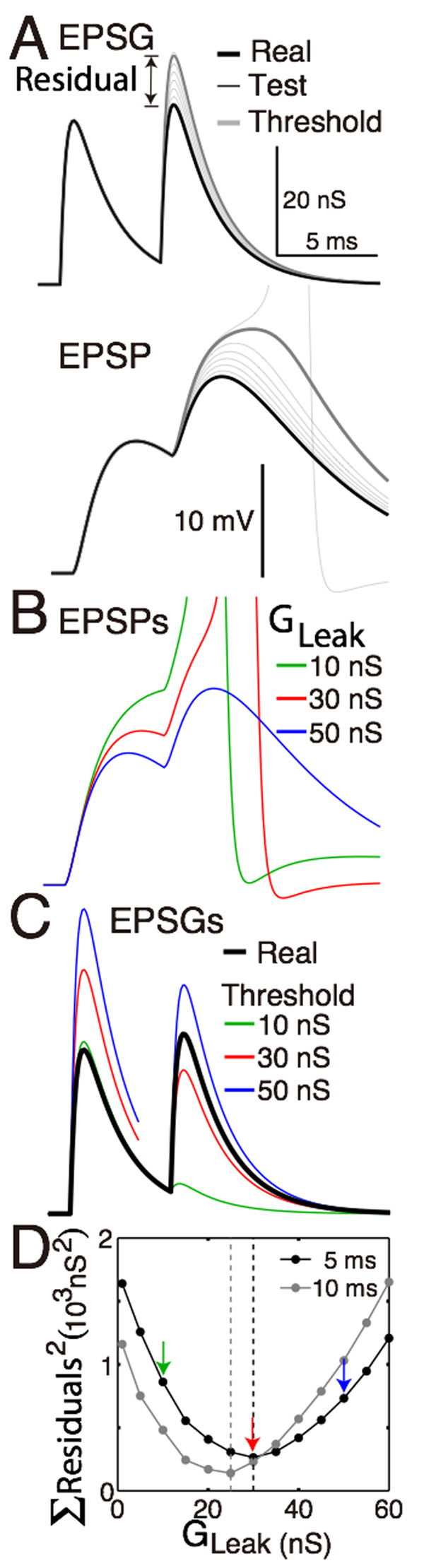
Finding the optimal homeostatic conductance. A neuron with only a leak conductance (reversal at -70 mV) and spike mechanism was simulated using NEURON software. **A.** The method of measuring distance from optimality. Top, the neuron received two EPSGs of equal amplitude (30 nS) separated by a 5 ms interval (thick black). At the time of each real EPSG, test ESPGs (thin black, shown only for the second) of varying amplitudes were applied to find the “threshold EPSG” (thick gray) for which the EPSP peak (bottom) is precisely at spike threshold. The “residual” is the difference in peak amplitude of the real and threshold EPSG, and it measures the distance of excitability from optimality. **B.** EPSPs generated by the real EPSGs in ‘A,‘ but with leak conductances of 10, 30, and 50 nS. **C.** Threshold EPSGs for the same three leak conductances. By comparing to the real EPSGs, it can be seen that although the 10 nS conductance best minimized the residual for the first EPSG, the sum of the two squared residuals is less for the 30 nS conductance. **D.** The sum of squared residuals was minimized by leak conductances of 30 and 25 nS in the case of 5 and 10 ms inter-EPSG intervals, respectively.

We have proposed that membrane excitability implements “predictive homeostasis” by predicting the amplitude of excitatory postsynaptic conductances (EPSGs) and generating a spike when EPSG amplitude exceeds the expectation. A spike would therefore represent a “positive prediction error.” “Excitability” measures distance from spike threshold and corresponds to the expectation. If the expectation is optimal, the peak of the excitatory postsynaptic potential (EPSP) will be precisely at spike threshold, and therefore the spike probability would be 0.5 and the spike output would be maximally sensitive to EPSG amplitude. Simultaneous recordings of retinogeniculate input and thalamocortical spike output support the theory by showing that spike probability is near 0.5 on average. This appears random to an external observer, but is maximally informative to the neuron. If the theory is correct, we should be able to predict the membrane properties of neurons given knowledge of the natural patterns of synaptic excitation that they receive. There are numerous temporal patterns of synaptic excitation in any neuron, and we believe that there are numerous homeostatic synapses and ion channels that contribute to predictive homeostasis. We would like to relate a specific pattern to a specific property of a homeostatic ion channel. We illustrate our approach by finding the optimal homeostatic leak conductance for a neuron that receives two EPSGs separated by 5 ms (Fig. 1). We propose that the optimal conductance is the one that minimizes the sum of the squared “residuals,” where the residual is the difference between the peak of the real EPSG and that of the “threshold EPSG” that would be required to cause an EPSP that reaches precisely to spike threshold (Fig. 1). Given that the tested intervals of 5 and 10 ms are short enough to allow temporal summation of the EPSPs (Fig. 1B), the optimal leak conductance will necessary be too high and too low at the time of the first and second EPSGs, respectively (Fig. 1C). Homeostasis could be better maintained by dynamic inhibitory conductances that increase following the onset of the first EPSG and thereby suppress the EPSP following the second EPSG. We propose that A-type K+ and GABA_A_ Cl- channels serve this function in real neurons. We plan to test this hypothesis by using the method described here to estimate the optimal densities or kinetics of these channels and comparing them with experimentally measured estimates.

